# Intention Concepts and Brain-Machine Interfacing

**DOI:** 10.3389/fpsyg.2012.00455

**Published:** 2012-11-09

**Authors:** Franziska Thinnes-Elker, Olga Iljina, John Kyle Apostolides, Felicitas Kraemer, Andreas Schulze-Bonhage, Ad Aertsen, Tonio Ball

**Affiliations:** ^1^Epilepsy Center, University Medical Center FreiburgFreiburg, Germany; ^2^Institute for Biology III, University of FreiburgFreiburg, Germany; ^3^Bernstein Center Freiburg, University of FreiburgFreiburg, Germany; ^4^Hermann Paul School of LinguisticsFreiburg, Germany; ^5^Juniata CollegeHuntingdon, PA, USA; ^6^Department of Philosophy and Ethics, Eindhoven University of TechnologyEindhoven, Netherlands

**Keywords:** BMI, BCI, action intention, intentional, philosophy of mind

## Abstract

Intentions, including their temporal properties and semantic content, are receiving increased attention, and neuroscientific studies in humans vary with respect to the topography of intention-related neural responses. This may reflect the fact that the kind of intentions investigated in one study may not be exactly the same kind investigated in the other. Fine-grained intention taxonomies developed in the philosophy of mind may be useful to identify the neural correlates of well-defined types of intentions, as well as to disentangle them from other related mental states, such as mere urges to perform an action. Intention-related neural signals may be exploited by brain-machine interfaces (BMIs) that are currently being developed to restore speech and motor control in paralyzed patients. Such BMI devices record the brain activity of the agent, interpret (“decode”) the agent’s intended action, and send the corresponding execution command to an artificial effector system, e.g., a computer cursor or a robotic arm. In the present paper, we evaluate the potential of intention concepts from philosophy of mind to improve the performance and safety of BMIs based on higher-order, intention-related control signals. To this end, we address the distinction between future-, present-directed, and motor intentions, as well as the organization of intentions in time, specifically to what extent it is sequential or hierarchical. This has consequences as to whether these different types of intentions can be expected to occur simultaneously or not. We further illustrate how it may be useful or even necessary to distinguish types of intentions exposited in philosophy, including yes- vs. no-intentions and oblique vs. direct intentions, to accurately decode the agent’s intentions from neural signals in practical BMI applications.

## Introduction

Intentions lie at the heart of human goal-directed behavior and have been debated for centuries in the philosophy of mind. Such fundamental issues have been discussed as the role of rational thought in intention formation (Bentham, 1781; Kant, 1785; Wittgenstein, 1953; Davidson, 1963; Kiverstein, 2006; Mele, 2007), and the temporal dynamics in and across distinct stages or kinds of intending (Searle, 1983; Pacherie, 2006). Various definitions of intention have been given, and a number of classifications have been proposed.

Broadly speaking, intention can be conceived of as a mental state in some way linked to phenomena such as decision, agency, desire, and belief (e.g., Anscombe, 1963; Goldman, 1970; Bratman, 1987). It is widely, though not universally, assumed that intention is causal to intentional action (Davidson, 1963). Theories differ with respect to the question whether intentions count as distinctive mental states (the non-reductive approach) or not (the reductive approach), see Pacherie (2002) for a review and Setiya (2007) and Bratman (2009) for a recent discussion. The exact nature and definition of intention are thus a matter of debate. Here we proceed from the influential definition of intention proposed by Bratman (1987). It relies on a superordinate category of “pro-attitudes,” which “play a motivational role” (1987, p. 15) in action. According to Bratman (1987), intentions and desires are distinctive mental states that fall into this category. A fundamental difference between the two is that intentions are “conduct-controlling” (1987, p. 16), whereas desires are “merely potential influencers of action” (1987, p. 16).

Owing to the advancements in neural-recording methodology over the last 50 years, various topographic, temporal, and semantic (content) manifestations of intentions in the human brain have been researched (Libet et al., 1983; Lau et al., 2004; Brass and Haggard, 2007, 2008; Haynes et al., 2007; Krieghoff et al., 2009; Bara et al., 2011) and are receiving further attention in cognitive neuroscience. The phenomenology and neurobiology of intentions are important to study for several reasons. A better understanding of causes and prerequisites for volitional behavior may aid objective evaluation of a person’s actions in ethical and legal contexts (Haggard, 2008; Schleim, 2008). Furthermore, such knowledge may help to treat patients with intention-related disorders, such as anarchic hand and Tourette’s syndromes (Haggard and Clark, 2003; Pacherie, 2007; Eddy et al., 2010; Edwards et al., 2011).

Conceptual input from the philosophy of intentions to other disciplines has previously proven useful. The belief-desire-intention model by Bratman (1987), for instance, was employed in computer science to develop the belief–desire–intention software model for programming intelligent agents (Rao and Georgeff, 1991). Similarly, philosophy may provide valuable input to the neuroscience of volitional action (Haggard, 2005; Mele, 2008; Pacherie and Haggard, 2010; Pacherie, 2011), and first attempts have recently been made to integrate philosophically-informed intention concepts into human neuroscience (Bara et al., 2011). Here, we propose that intention concepts from the philosophy of mind may be also usefully adopted by the emerging field of brain-machine interfacing (BMI) research and technology. In particular, we argue that intention concepts are important for BMIs utilizing higher-order intention-related brain activity, in contrast to BMIs that are based solely on inference of low-level movement parameters. The also widespread P300 BMI approaches as well as those based on learned self-regulation of brain signals remain outside the scope of this article. Recent reviews on these topics can be found in Fazel-Rezai et al. (2012) and Wolpaw et al. (2002), respectively.

The structure of this article is as follows. In Section “Insights into Intentions from Cognitive Neuroscience,” we review current neuroscientific literature on intentions, and outline the core areas involved in intention-related processing in humans. In Section “Current Approaches to Brain-machine Interfacing,” we address the basic principles that are currently employed in BMI-based restoration of motor and communication functions. In Section “Philosophical Taxonomies of Intentions and their Relevance to BMI,” we summarize some influential philosophical notions and taxonomies of intentions, and illustrate their potential relevance for neuroscientific research in general and in particular for BMI. Finally, we draw conclusions and provide an outlook for future studies in Section “Conclusions and Outlook.”

## Insights into Intentions from Cognitive Neuroscience

**Figure 1:**
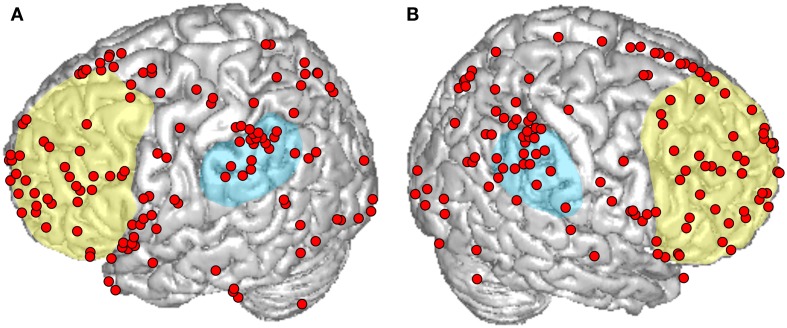
**An overview of cortical responses reported in recent functional magnetic resonance imaging (fMRI) and positron emission tomography (PET) studies that explicitly aimed at investigating intentions in healthy subjects**. Peaks are plotted on a standard brain from SPM8 on **(A)** the left and **(B)** right hemisphere. The approximate locations of the prefrontal and the inferior parietal cortex are indicated in yellow and blue, respectively. Reported intention-related peaks in both hemispheres exhibit a widespread spatial distribution across the frontal, parietal, occipital, and temporal lobes.

Interest in intention-related brain signals has grown in neuroscience over the last several decades. In their early electroencephalography (EEG) readiness-potential study, Libet et al. (1983) reported that cerebral activity before initiation of self-paced movements precedes the conscious intention to move over several 100 ms. The observed temporal differences led these authors to conclude that initiation of voluntary actions can begin unconsciously, and it is only some time later that we become aware of an intention to move. Although the reported findings and their interpretation were highly controversial (e.g., Keller and Heckhausen, 1990; Snyder et al., 1997; Haggard and Eimer, 1999), the article by Libet et al. (1983) contributed to the development of a vivid discussion about the nature of human free will, agency and voluntary movement, and was followed by a large amount of experimental studies and opinion articles concerning the neural correlates of intentional action. Consecutive research identified a widespread distribution of neural locations in the frontal, parietal, and even temporal lobes (Figure 1), arranged in extended cortical networks for intention-related processing (Haggard, 2008).

One cortical location that has been repeatedly activated in studies on intention-related processing is the posterior parietal cortex (PPC), in particular its inferior part (Figure 1). Initial evidence for the contribution of this region to intentional control comes from single-cell recordings in monkeys. Specifically, the parietal reach region (PRR) and the lateral intraparietal area (LIP) have been shown to exhibit effector-specific neuronal activity in delayed saccadic and reaching tasks (Andersen and Buneo, 2002; Quian Quiroga et al., 2006; Cui and Andersen, 2007; Andersen and Cui, 2009), suggesting that the PPC can convey neuronal information about what the animal intends to do (Snyder et al., 1997). In humans, involvement of parietal regions in intention-related processing was observed using electrical stimulation, which elicited a reported “urge to move” without consecutive execution (Assal et al., 2007; Desmurget et al., 2009), and in lesion studies showing that awareness of an intention to move is abnormal in patients with damage to the parietal cortex (Sirigu et al., 2004; Assal et al., 2007). Parietal contributions to intention encoding were also confirmed by a number of functional magnetic resonance imaging (fMRI) studies. In prospective memory tasks, both lateral and medial parietal regions showed increased blood-oxygen-level dependent (BOLD) responses that stretched from the precuneus into the anterior and posterior cingulate cortices, the intraparietal sulcus, and inferior parietal regions (Burgess et al., 2001; den Ouden et al., 2005; Eschen et al., 2007; Haynes et al., 2007; Soon et al., 2008; Gilbert, 2011; Benoit et al., 2012; Momennejad and Haynes, 2012). Investigations of non-delayed self-initiated movements reported similar neural responses in the inferior parietal lobe (Ball et al., 1999; Farrer et al., 2008; Krieghoff et al., 2009), in the intraparietal sulcus (Lau et al., 2004; Gallivan et al., 2011a,b), and in the anterior cingulate cortex (Cunnington et al., 2006; Mueller et al., 2007; Krieghoff et al., 2009), as opposed to externally triggered movements, which elicit no, or only attenuated activations in these regions (Jahanshahi et al., 1995; Jenkins et al., 2000; Mueller et al., 2007; Hoffstaedter et al., 2012).

In addition to parts of the parietal cortex, the prefrontal cortex (PFC, Figure 1) has been activated in many intention-related studies. Delayed intention paradigms revealed lateral and medial PFC responses, mostly in rostral prefrontal areas (Burgess et al., 2001; den Ouden et al., 2005; Simons et al., 2006; Poppenk et al., 2010; Gilbert, 2011; Benoit et al., 2012), whereas non-delayed intention experiments showed activity in the dorsal medial and lateral prefrontal regions (Lau et al., 2004; Cunnington et al., 2006; Rushworth, 2008; Gallivan et al., 2011a,b; Rosenberg-Katz et al., 2012). The frontopolar cortex (BA10) was suggested to represent a gateway mechanism for orienting attention toward external and internal stimuli, and to play a critical role in the encoding and storage of future intentions (den Ouden et al., 2005; Haynes et al., 2007; Soon et al., 2008; Uretzky and Gilboa, 2010). In accordance with the latter, clinical evidence shows that lesions in this area lead to the impaired ability to keep future intentions in mind for later execution (Burgess et al., 2001).

Intention-related information is also thought to be present in higher-order motor areas, including the supplementary motor area (SMA; Eccles, 1982; Fried et al., 1991, 2011; Jahanshahi et al., 1995; Ball et al., 1999; Jenkins et al., 2000; Lau et al., 2004; Soon et al., 2008; Hoffstaedter et al., 2012; Momennejad and Haynes, 2012), the pre-SMA (Lau et al., 2004, 2006; Cunnington et al., 2006; Mueller et al., 2007; Nachev et al., 2007), and in the dorsal and ventral premotor regions (Cunnington et al., 2006; Pesaran et al., 2006; Eschen et al., 2007; Gallivan et al., 2011a,b; Hoffstaedter et al., 2012). Since activity in the SMA and the pre-SMA typically occurs early and precedes movement execution (Fried et al., 2011), and considering that activation in the pre-SMA has been observed in relation to own intentions as opposed to own movements (Lau et al., 2004), these areas may contribute to intentional processes during preparation for action (Passingham et al., 2010).

Finally, the anterior insular cortex has been co-activated with several aforementioned areas in studies on intention encoding in the human brain (Jahanshahi et al., 1995; Mueller et al., 2007; Krieghoff et al., 2009; Hoffstaedter et al., 2012). Insular activation has been proposed to subserve evaluation of possible consequences of intentional actions (Brass and Haggard, 2010).

In addition to these core areas, intention-related activity has been reported in many other brain regions with a widespread distribution as shown in Figure 1, which presents an overview of cortical activation foci reported by recent neuroimaging studies that explicitly aimed at investigating intentions in healthy subjects. Using these criteria, we identified 22 studies (Burgess et al., 2001; Lau et al., 2004, 2006; den Ouden et al., 2005; Cunnington et al., 2006; Simons et al., 2006; Eschen et al., 2007; Haynes et al., 2007; Mueller et al., 2007; Farrer et al., 2008; Soon et al., 2008; Krieghoff et al., 2009; Poppenk et al., 2010; Gilbert, 2011; Hashimoto et al., 2011; Okuda et al., 2011; Benoit et al., 2012; Gilbert et al., 2012; Hoffstaedter et al., 2012; Momennejad and Haynes, 2012; Rosenberg-Katz et al., 2012) reporting a total amount of 303 cortical and subcortical intention-related peaks.

We performed an activation likelihood estimate analysis (ALE; as described in Mutschler et al., 2009) of these studies to statistically detect brain regions with responses that occur red reproducibly. This revealed only two clusters with significant ALE (*p* < 0.05, FDR-corrected). Both of them were located in the SMA (assigned to Brodmann area 6 with maxima at MNI coordinates −2; 16; 54 and −4; 14; 50, and with respective probabilities of anatomical assignment of 40 and 50% (Eickhoff et al., 2005). There may be several reasons why only these clusters were significant. First, the number of studies satisfying our strict selection criteria was limited. Future meta-analyses based on larger samples may reveal additional foci of reproducible neural responses. Second, as argued in Brass and Haggard (2008, p. 319), the spread of neural activity seen in neuroscientific literature on intentions may be because “intentional action has been treated as a unitary concept within neuroscience, even though experimental studies may focus on any of a number of different aspects of intentional action.” Meta-analyses distinguishing different types and aspects of human intention may be necessary to reveal more reproducible neural responses. Applying the same idea to the field of BMI research, in the following section we discuss to what extent it may be useful or even necessary to integrate different types as well as temporal and semantic aspects of intentions to develop safe and efficient real-life BMI applications.

## Current Approaches to Brain-Machine Interfacing

**Figure 2:**
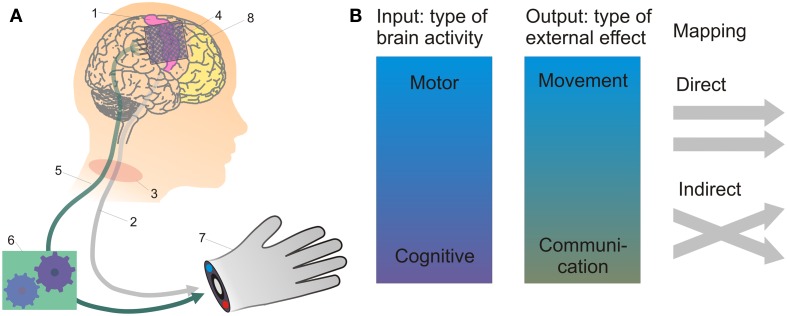
**The working principle and current approaches of BMI**. In **(A)**, BMI-based control of a prosthetic hand by using recordings of brain activity is depicted. The projections from the motor cortex (1) constituting the cortico-spinal pathways (2) may be disrupted, e.g., by spinal-cord injury (3). Then, suitable electrodes (4) can be used to record persistent motor-cortical activity, which is transmitted by a technical connection (5; either wire-based or wireless) to a decoder (6) extracting control signals for an external actuator (7). If the primary motor cortex is destroyed, such as due to stroke, cognitive control signals may still be recorded from alternative areas such as the prefrontal cortex (8). As summarized in **(B)**, neural control signals may thus range from low-level motor signals, such as related to movement direction or velocity, to more high-level cognitive signals related to abstract action goals, subjective preferences, and intentions. Output signals may be used to restore movement (e.g., of an external actuator) or communication. The input-output mapping can be realized in a direct way, e.g., if right- and leftward movement-related neural activity controls the respective right- and leftward movements of an effector, the intention to grasp a cup is directly translated into the corresponding grasping action, or the intention to say the word “hello” is directly translated into speech. Indirect strategies would be, for example: using imagined leg vs. tongue movements to control right- vs. leftward movements of a robotic arm.

Brain-machine interfaces allow humans to control technical devices through direct recordings of brain activity. To this end, the device – either intracranial (brain-implanted) or extracranial (fixed on the person’s skull) – measures the brain activity of an agent, interprets (“decodes”) the agent’s intended action, and sends the corresponding execution command to an artificial effector system, such as a computer cursor, a prosthetic limb, or a wheelchair (Figure 2A). First clinical trials have demonstrated the success of the BMI principle for restoration of movement (Hochberg et al., 2006, 2012) and communication (Birbaumer et al., 1999; Guenther et al., 2009) in paralyzed individuals.

Brain-machine interfacing approaches may be categorized by the type of brain signal used (single-neuron activity, neural-population signals, etc.) and the invasiveness of the recording technique (Waldert et al., 2009). To assess the potential importance of intention concepts for practical BMI purposes, we shall characterize BMIs according to (i) the type of neural activity “input signal” used to decode information, (ii) the type of external output that is generated, and (iii) the kind of mapping between input and output (Figure 2B).

Regarding neural input, an important distinction can be made between BMIs using “low-level” motor signals, such as changes in neural activity related to movement direction or velocity, and BMIs utilizing “higher-level,” cognitive signals. These may relate to subjective preferences or abstract action goals (Musallam et al., 2004). Between these extremes, there is a spectrum of more or less abstract/cognitive control signals that have been used, or are at least in principle usable, for BMI applications. Such signals can reflect that action plans are represented at different levels of abstraction in the brain (Bonini et al., 2011). Many current BMIs use low-level motor control signals recorded from primary or secondary motor areas (Hochberg et al., 2006, 2012; Moritz et al., 2008; Pistohl et al., 2012). BMIs based on this approach, however, still have much room for improvement in terms of decoding accuracy, especially in complex motor tasks. These and other challenges of present BMI technology are addressed further in a recent review by Schalk and Leuthardt (2011).

Cognitive signals may help to make BMI control more accurate. Based on the decoded abstract goals or intentions, intelligent autonomous external devices can perform lower-level computations, such as trajectories, that are necessary to achieve movement goals (Musallam et al., 2004). This approach may serve to lower bandwidth requirements for BMIs. Furthermore, if the brain structures that allow decoding movement-related signals (e.g., the primary motor cortex) are dysfunctional due to pathological processes, cognitive neural control signals, such as action goal- and intention-related activity from higher-order brain regions including premotor, posterior parietal, and PFC, may be used to substitute.

On the output side, the information decoded from either low- or high-level signals may be harnessed to generate movement (of a screen cursor, robotic arm, wheelchair, or even of a patient’s own limbs via electrical stimulation of the muscles) or communication signals (as ringing an alarm bell, controlling a spelling device for writing, or synthesizing acoustic speech). Again, there are intermediate cases, such as if signals related to attempted right- and left-hand movement (a motor signal) were used to select a part of the alphabet or a letter in a spelling device (a communication output).

Different strategies may be used to map the input (brain) signal to the (externally-directed) output signal. We refer to those that aim to restore movement or speech functions with neural signals underlying the same function as “direct.” For instance, a direct motor BMI would use brain signals related to left- vs. rightward movements to generate left- vs. rightward movements of an effector (Leuthardt et al., 2006; Milekovic et al., 2012). A direct speech BMI may use neural signals related to the respective phoneme (Blakely et al., 2008; Guenther et al., 2009; Pei et al., 2011), word (Kellis et al., 2010), semantic content (Wang et al., 2011), and context-dependent style (Derix et al., 2012) to generate matching speech output. Thus, neural activity related to the intended word “hello” would be decoded to spell “hello” in the BMI output (Kellis et al., 2010). In contrast, indirect approaches rely on neural input from tasks or modalities not directly related to output. For example, imagined leg vs. tongue movements may be used to control right- vs. leftward movements of a robotic arm. On this principle, Leuthardt et al. (2011) recently achieved BMI-based one-dimensional motor control using input signals related to production of overt and imagined phonemes. Indirect approaches have been widely used in non-invasive EEG-based BMI studies, since it is possible to select arbitrary tasks inducing highly distinctive global topographic EEG patterns, which can be robustly classified for BMI control.

The importance of intention-related brain signals and the potential role of intention concepts may vary depending on the BMI approach. A BMI based on low-level motor control signals may, at least to a certain extent, work without any such high-level information as intention-related signals. For approaches which do tap into intention-related processes in the brain, however, it may be useful or even necessary to take well-informed intention concepts into account, especially given a direct framework, i.e., if intentions are to be directly translated into the intended action.

Risks due to misinterpretation of neural control signals would be greatest for BMIs with effectors such as robotic arms or wheelchairs. In such applications, decoding of higher-order information with respect to the final goal of action as a whole may be a useful safety precaution, even if they primarily rely on low-level motor signals. In summary, intention concepts appear most relevant for *direct BMIs using cognitive neural control signals*, with both movement and speech output, but intention-related information may also constitute an auxiliary information channel for other types of BMIs.

## Philosophical Taxonomies of Intentions and Their Relevance to BMI

A properly designed intention-based BMI device should be able to clearly distinguish between different types of intentions. For instance, a patient using a BMI to steer a wheelchair may intend to turn right in a few seconds, right now, or next Wednesday, and the wheelchair must be sensitive to this temporal difference. At first glance, this distinction seems fairly trivial. Yet the question arises: How many different kinds of intentions can be identified by their temporal characteristics? And how are different types of intentions organized in time, that is, what are their individual dynamics, mutual transitions, and interactions? A number of intention theories (Searle, 1983; Brand, 1984; Bratman, 1987; Pacherie, 2006) have addressed the issue of timing and elaborated on various aspects of future- vs. present-oriented intentions.

Among other questions related to rational action, Bratman (1987) addressed differences between future- and present-directed intentions. According to his conceptual framework, future-directed intentions are formed prior to action and represent the product of deliberation whether or not to act in a certain way. An example is a future-directed intention to leave for Boston in April that has been formed in January (Bratman, 1987). In contrast, present-directed intentions inherit plans from future-directed intentions, and implement them in a current situation of action. Thus, if one has a future-directed intention to go to Boston in April, a present-directed intention may be to take a particular route that day and turn while driving to the airport, whereby the agent advances to complete his global plan. According to Bratman (1987), these two types of intentions are formed based on one’s desires and beliefs as to whether the action in question is in some way beneficial and necessary to the conscious agent.

**Figure 3:**
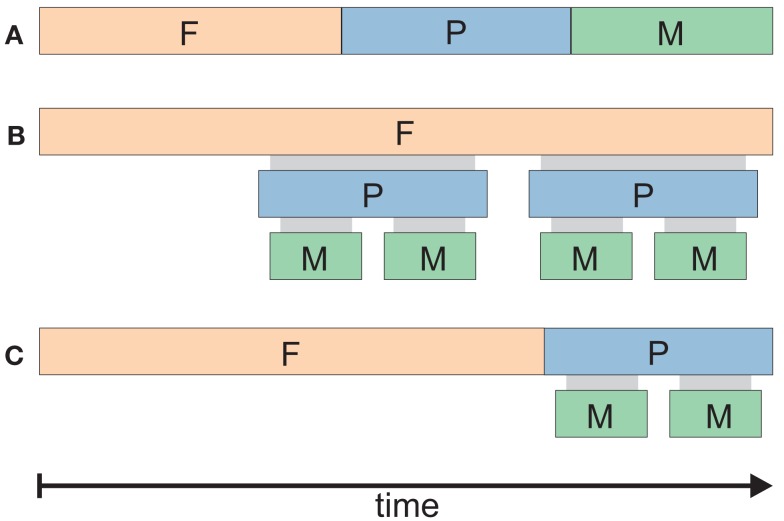
**(A)** Schematic representation of sequential **(A)** vs. hierarchical **(B)** vs. **(C)** mixed models of intention organization in time. Three different scenarios are depicted for the case of a threefold intention concept, roughly corresponding to the F-, P-, and M- intentions proposed by Pacherie (2006). The durations of F-, P-, and M-intentions are depicted by colored bars. A mixture of hierarchical and sequential relations is shown in **(C)**. Which of these different scenarios is true in a given situation would have important consequences for attempts to decode intentions; for example, the detection of an F-intention would rule out the simultaneous presence of the corresponding P- and M-intentions in the purely sequential **(A)** but not in the hierarchical **(B)** model. Note that Pacherie’s concept favors **(C)**, particularly in her recent work (Pacherie, 2008).

In a more recent philosophical paper, Pacherie (2006) adopted this terminology, referring to present- and future-directed intentions as P- and F-intentions, respectively. We will use these abbreviations from here on, also in cases where we do not refer to the specific theories by Pacherie (e.g., parts of Figure 3). In addition to these two types of intentions, Pacherie (2006) proposed a third category, the so-called motor-, or M-intentions, which inherit goals from present-directed intentions and initiate a motor program satisfying the spatial and temporal demands for action realization (Pacherie, 2006). One main reason for introducing this additional category was the consideration that, whereas both P- and F-intentions are subject to strong rationality constraints (Bratman, 1987), not all voluntary actions require deliberation. Examples are such automated, routine actions as a smoker reaching for a pack of cigarettes and realizing that she is doing it already in the process of reaching (Pacherie, 2006), or a person who unlocks his office door by mere habit of doing so every morning (Mele, 2007).

The F-, P-, and M-intentions have distinct functional roles. Based on Bratman’s account of F-intentions (1987), Pacherie (2006, p. 3) assumes that F-intentions serve as “terminators of practical reasoning about ends, prompters of practical reasoning about means and plans, and intra- and interpersonal coordinators.” The conscious P-intentions ensure “higher-level guidance and monitoring,” whereas “lower-level guiding and monitoring functions should properly be assigned to M-intentions” (Pacherie, 2006, p. 5). But what is the exact *temporal* organization of intentions – is it hierarchical, or do intentions unfold sequentially?

A sequential model of intentions (illustrated in Figure 3A) would assume that the F-, P-, and M- intentions precede each other, and one type of intention stops once it has passed its goal onto the next type which directly follows. A hierarchical model (Figure 3B), though, would assume that all three types of intentions overlap in time and govern one another in synchrony.

Concerning F-intentions, Pacherie (2006, p. 4) states that they are “in principle detachable from the agent’s current situation and [are] indeed commonly detached from it,” and that “insofar as they are temporally separated from the action, [F-intentions] make no direct contribution to the experience of acting” (Pacherie, 2006, p. 14). In contrast, “P-intentions and M-intentions are both simultaneous with the action that they guide” (Pacherie, 2006, p. 14). While these views were still at least to some extent consistent both with models B and C (Figure 3), in her more recent work, Pacherie (2008) sides more clearly in favor of a mixed sequential-hierarchical model (Figure 3C), in which F-intentions occur before P- and M-, and the latter two types of intentions take place simultaneously.

The temporal model of intentions we inferred based on work by Pacherie (2006); Figure 3C), as well as the other two models (A and B in Figure 3), may be a useful reference to interpret observations of action- and intention-related neural activity at different temporal scales. Furthermore, these temporal models of intentions may entail important consequences for attempts to decode intentions. For example, the detection of an F-intention would rule out the simultaneous presence of the corresponding P- and M-intentions in the purely sequential but not in the hierarchical model. The decoding problems for BMI devices will differ accordingly.

If our experience of acting is directly governed by P- and M-, but not by F-intentions (Pacherie, 2006), it seems plausible that the neural correlates of F-intentions could considerably differ from that of both P- and M-intentions. Future-oriented intentions in neuroimaging studies have been mostly investigated in the context of prospective memory (Burgess et al., 2001; den Ouden et al., 2005; Simons et al., 2006; Eschen et al., 2007; Poppenk et al., 2010; Gilbert, 2011; Hashimoto et al., 2011; Okuda et al., 2011; Benoit et al., 2012; Gilbert et al., 2012; Momennejad and Haynes, 2012), and action intentions in a current situation of action have also been the focus of recent research (Cunnington et al., 2006; Gallivan et al., 2011a,b), revealing partially overlapping neural effects. A contrastive investigation into these two paradigmatic frameworks in the literature may be of interest in future studies including meta-analyses, and can be expected to reveal a topographically differential distribution of neural effects related to future- vs. present-directed intentions.

The idea that F-intentions are indeed formed prior to P-intentions, and do not directly contribute to the experience of action (Pacherie, 2006), may have important implications in the context of BMI. As the term implies, F-intentions deal with prospective plans (Bratman, 1987; Pacherie, 2006), so they may be used as possible coordinators for the fine-tuning of an intention decoder. Knowledge of the global plan of an intention is crucial for understanding action orientation as a whole (Bara et al., 2011). F-intentions could thus serve to improve the accuracy of a BMI, and ensure goal-oriented guidance of BMI-mediated action.

To differentiate the consciously experienced P-intentions from M-intentions, which are not subject to rationality constraints (Pacherie, 2006), is also essential for safe and efficient BMI-based restoration of motor functions. According to Pacherie (2006), p. 9), when M-intentions occur without P-intentions, they initiate “a competition among motor programs, with the program showing the strongest activation being triggered,” such as in the above-mentioned example of reaching for a pack of cigarettes (Pacherie, 2006), possibly even when smoking is prohibited. BMI-based realization of M-intentions that do not inherit their goal from a P-intention may be, in some cases, dangerous, and in conflict with a higher-level “no-intention” (see below). On the other hand, unconscious intentions may play an important role in performance of automatic actions when no time for deliberation is available, e.g., when driving a car. Thus, there seems to be no general solution as to which cases of “isolated” M-intentions should be executed. Future research can be expected to shed more light on this issue.

Philosophical accounts may provide further theoretical ground for BMI research in their distinction between intentions to perform and intentions not to perform an action (Harman, 1986; Bratman, 1987; Setiya, 2011). For the sake of brevity, we suggest the terms *yes*- vs. *no-intentions*. Confusing these phenomena would severely compromise the safety of a BMI device. Intentional inhibition, which may be considered as a no-intention, has been investigated in human neuroscience (Brass and Haggard, 2007, 2008; Kühn and Brass, 2009; Kühn et al., 2009), identifying responses in the dorsal fronto-medial cortex distinct from areas implicated in what we refer to as yes-intentions (Brass and Haggard, 2007; Kühn et al., 2009). These findings may provide valuable information for emergent BMI technology, particularly to accurately interpret intended action vs. intended restraint.

Another distinction relevant to BMI is that between *direct* and *oblique* (i.e., indirect) intentions, as proposed by the English philosopher Jeremy Bentham in the late eighteenth century (1781, repr. 2000). Bentham (1781, p. 70) explains this distinction through a discrete relation between will, actions and consequences:

“A consequence […] may be said to be directly or lineally intentional, when the prospect of producing it constituted one of the links in the chain of causes by which the person was determined to do the act. It may be said to be obliquely or collaterally intentional, when although the consequence was in contemplation, and appeared likely to ensue in case of the acts being performed, yet the prospect of producing such consequence did not constitute a link in the aforesaid chain.”

Bentham (1781, p. 71) exemplifies his account of direct and oblique intentions departing from a historical case of William II, king of England being deadly wounded by the nobleman Walter Tyrrel during a hunt. The circumstances of this incident remained unclear. According to Bentham, there are several possible ways to evaluate the intentionality of Sir Tyrrel’s actions. One imaginable scenario is that the king is riding close to a stag, and Sir Tyrrel shoots his arrow with the aim to kill the stag; he is convinced that the shot is not dangerous to the king. The killing in this case occurs by accident and Bentham classifies it as unintentional. A second possibility is that Sir Tyrrel aims to kill the stag and shoots at it, although he is aware that the shot is as likely to kill the king as the stag. If Sir Tyrrel’s shot kills the king in this scenario, his actions can be regarded as obliquely intentional. A third possibility is that Sir Tyrrel hates the king and shoots with no other aim than to kill him. In this latter case, Sir Tyrrel’s actions classify as directly intentional. Thus, direct and oblique intentions differ as to whether the outcome of the action is actively sought-after (direct intentions) or a foreseeable “side effect” (oblique intentions).

Initially, this classification was developed to evaluate the degree of responsibility for harmful actions in the legal context. However, we believe that the distinction between direct and oblique intentions may be also of relevance to the emerging field of BMI. Imagine a person with a BMI-controlled prosthetic arm is having breakfast, and moves to reach a piece of bread, just behind his cup of coffee. The person aims to reach the bread (direct intention) and not to touch or topple the cup of coffee, although he understands that these consequences may occur (oblique intention). It is important that a BMI relying on inference of intentions does not confuse direct with oblique intentions, and gives priority to the execution of the former, to perform its user’s effective wishes. To our knowledge, this direct vs. oblique distinction has not been investigated in cognitive neuroscience, and it is currently unclear which neural substrates support these different kinds of intentions.

Another important secernment is between what we call *mere urges* and *action intentions*. A review of the existing literature distinguishing intentions, urges, and desires, however, is beyond the scope of the present article (for literature on these distinctions, see Johnston, 2001; Mele, 2007). An urge may be phenomenologically described as a strong impulse toward an action. Urges are typically stimulus-evoked, such as an urge to scratch evoked by an itch or an urge to cry by a sad situation or thought. Urges may be delineated from desires in that desires have an evaluative element, i.e., the object of the desire is “desirable” and “good” in some way, while urges lack this (Scanlon, 1998, 2008). Within neuroscience, the neural underpinnings of urges have been, until now, most extensively investigated in the specific context of drug craving (Maas et al., 1998; Childress et al., 1999) and in electrical stimulation studies (Fried et al., 1991).

If the driving force of an urge becomes overwhelming, it may result in an “urged action” – even against one’s intentions. However, it is a fundamentally important aspect of human behavior that urges can be controlled, and blocked if necessary. Here, we refer to an urge without any associated intention to perform an action as a *mere urge* – in contrast to an urge toward an action that is actually intended (following a similar idea as Pockett and Miller (2007), who distinguish a mere urge from an actual decision).

A BMI should likewise distinguish mere urges from action intentions. The relevance of this distinction becomes clear from the examples that follow. Imagine that a person with a BMI-controlled bionic arm becomes as angry at a rude conversation partner as to experience aggressive urges, such as to punch him for the offense. Punching the offender, however, does not correspond to the person’s actual intentions. In this and similar cases, it is vitally important that the BMI device does not translate the mere urge into motor performance.

Another likely situation is that a person with a BMI-controlled arm is bitten by a mosquito and experiences an urge to scratch the bite. The person is aware that to scratch may further hurt the skin and make the itch even worse, so he decides to refrain from scratching. To prevent the execution of such unintended, and potentially dangerous movements, it will be necessary for the BMI to keep mere urges and intentions apart.

Whether the action is other- or agent-directed, an important question regarding misinterpretation of mere urges and intentions by BMI technology is: If someone is hurt in such a scenario, is the user responsible, or the manufacturer of the device? It seems plausible that a mere urge as defined above is not morally significant, and that a BMI application must be able to distinguish it from an action intention.

## Conclusions and Outlook

Intention is often treated as a unitary concept in neuroscientific research (Brass and Haggard, 2008). Philosophy may help to discern types of intentions, and give a more differential account. Studies of intentions in neuroscience that have explicitly used philosophically-informed concepts are however rare at present. As a notable exception, Bara et al. (2011) investigated, using fMRI, several types of future intentions, namely, private, prospective social, and communicative intentions, incorporating classifications proposed by Searle (1983), Bratman (1987), Pacherie and Haggard (2010), as well as by Tomasello (2008). Bara et al. (2011) found that all of these intention types were associated with activation in the right temporo-parietal junction and the precuneus; the activity in the left temporo-parietal junction and the medial PFC, however, was specifically observed in relation to prospective social intentions.

In addition to these first steps toward incorporating intention concepts from philosophy into basic neuroscience (Bara et al., 2011), philosophical accounts may contribute to the development of BMIs based on cognitive control signals, as has been argued above. Examples in the previous section illustrate how a better understanding of temporal properties of intentions is important for safe and efficient BMI performance. Together, the reviewed literature strongly suggests that it would be particularly important to study the neural basis of intentions (i) at different temporal scales (Searle, 1983; Brand, 1984; Bratman, 1987; Pacherie, 2006) and (ii) taking into account the issue of hierarchical vs. sequential organization of intentions in time (Pacherie, 2006, 2008).

Further research to improve BMI may also benefit from understanding qualitative differences between various types of intentions that have been proposed in philosophy, including yes- vs. no-intentions (Harman, 1986; Bratman, 1987; Setiya, 2011), and direct vs. oblique intentions (Bentham, 1781). Only a few neuroscientific studies so far have investigated intentional inhibition of actions (Brass and Haggard, 2007, 2008; Kühn and Brass, 2009), which is apparently analogous to a no-intention, and of yet we are not aware of any study dedicated to the direct vs. oblique distinction.

The temporal and semantic components of intention seem to be strongly related, as the degree of content abstraction is generally higher in future- compared to present-directed intentions (Searle, 1983; Mele, 1989; Pacherie, 2006). Nevertheless, it is also imaginable that intentions directed at the same time in the future may still vary with respect to their level of abstraction. For instance, one may intend to go on a holiday next summer, or one may intend to go on a holiday to the same nice hotel in Ronda next summer. Conversely, intentions with different temporal targets may exhibit a higher degree of content similarity relative to other intentions with the same time to action execution. Content abstraction may hence be important to include into further empirical research on intention dynamics as an additional, at least partially independent factor. Insights from such investigations may be useful to determine the exact onset of an intended action in BMI-based movement restoration.

Alongside philosophical intention taxonomies, conceptual input from psychology and cognitive science may be of value. For instance, a conceptual framework incorporating the “what,” “when,” and “whether” components of intentional action has been proposed (Brass and Haggard, 2008) and neuroscientifically investigated (Brass and Haggard, 2007; Mueller et al., 2007; Krieghoff et al., 2009; Kühn and Brass, 2009; Kühn et al., 2009; Obhi et al., 2009; Hoffstaedter et al., 2012; Momennejad and Haynes, 2012). Further hallmark questions to be addressed in future interdisciplinary research are: (i) Can philosophical intention taxonomies be used as direct input for BMI studies, or do they first need to be operationalized to be applicable to neuronal data? (ii) What are the correspondences and differences between philosophical and psychological concepts of human intent? In addressing these questions, not only may neuroscience and BMI research benefit from cooperation with philosophy, but also vice versa: insights into the biological plausibility of different aspects and types of intentions proposed in the philosophy of mind may provide valuable empirical feedback, thereby closing the loop from neuroscience to philosophy.

## Conflict of Interest Statement

The authors declare that the research was conducted in the absence of any commercial or financial relationships that could be construed as a potential conflict of interest.
